# Could FaRP-Like Peptides Participate in Regulation of Hyperosmotic Stress Responses in Plants?

**DOI:** 10.3389/fendo.2014.00132

**Published:** 2014-08-14

**Authors:** François Bouteau, Yann Bassaglia, Emanuela Monetti, Daniel Tran, Sandra Navet, Stefano Mancuso, Hayat El-Maarouf-Bouteau, Laure Bonnaud-Ponticelli

**Affiliations:** ^1^Sorbonne Paris Cité, Institut des Energies de Demain, Université Paris Diderot, Paris, France; ^2^LINV-DiSPAA, Department of Agri-Food and Environmental Science, University of Florence, Sesto Fiorentino, Italy; ^3^Muséum National d’Histoire Naturelle, DMPA, Sorbonne Universités, UMR BOREA MNHN-CNRS 7208-IRD 207-UPMC-UCBN, Paris, France; ^4^Faculté des Sciences and Technologies, Université Paris Est Créteil-Val de Marne (UPEC), Créteil, France; ^5^Sorbonne Paris Cité, Paris Interdisciplinary Energy Research Institute (PIERI), Université Paris Diderot, Paris, France; ^6^UPMC UMR7622-IBPS, Paris, France

**Keywords:** *Arabidopsis thaliana*, drought, FaRP-like peptides, osmotic stress, stomata

## Abstract

The ability to respond to hyperosmotic stress is one of the numerous conserved cellular processes that most of the organisms have to face during their life. In metazoans, some peptides belonging to the FMRFamide-like peptide (FLP) family were shown to participate in osmoregulation via regulation of ion channels; this is, a well-known response to hyperosmotic stress in plants. Thus, we explored whether FLPs exist and regulate osmotic stress in plants. First, we demonstrated the response of *Arabidopsis thaliana* cultured cells to a metazoan FLP (FLRF). We found that *A. thaliana* express genes that display typical FLP repeated sequences, which end in RF and are surrounded by K or R, which is typical of cleavage sites and suggests bioactivity; however, the terminal G, allowing an amidation process in metazoan, seems to be replaced by W. Using synthetic peptides, we showed that amidation appears unnecessary to bioactivity in *A. thaliana*, and we provide evidence that these putative FLPs could be involved in physiological processes related to hyperosmotic stress responses in plants, urging further studies on this topic.

## Introduction

Most of the living organisms from bacteria to metazoans, fungi, and plants have to face hyperosmolarity (i.e., an external osmolarity that is higher than the physiological range) during their lifetime, and the establishment of an appropriate response can be a matter of life or death. Whatever the cell types, they are generally able to counteract volume perturbations following a shift in extracellular osmolarity by rapidly modulating the activities of their plasma membrane ion transport systems (1, 2). Several major hormones that respond to osmotic stress have been identified in metazoans, vertebrates to arthropods, and plants (3–9), but it is only more recently that the importance of small peptides in different regulatory mechanisms has been pointed out in metazoans and plants (10, 11). In numerous metazoans (mollusks, annelids, nematodes, and vertebrates), peptides belonging to the FMRFamide-like peptides (FLPs) family have been shown to participate in osmoregulation (12, 13). Moreover, FLPs were shown to target various ion channels, among them the membrane sodium channels, such as the amiloride-sensitive FMRFa-activated sodium channel (FaNaCh) in invertebrates (13), or the structurally related acid-sensing sodium channels (ASICs) in vertebrates (14). These ligand-gated or pH sensitive-Na^+^ channels are involved in Na^+^ permeability and associated water transport, which makes them critical determinants of cell volume regulation (14). In sensory neurons of *Aplysia*, chloride currents are evoked by FMRFa via the cGMP cascade (15). In the same type of neurons, FMRFa also modulates the probability of opening and closing of S-type K^+^ channels, a stretch-activated channel involved in response to osmotic shock (16).

For plant, drought-induced osmotic stress and salinity represent some of the major constraints that adversely affect growth, development, and biomass production. Numerous cellular responses and proteins have been reported to be conserved between plant and animal cells (17–19). Among them are ion channels (20), and their involvement in response to hyperosmotic stress (2, 21–23). Recent works implicate small signaling peptides in developmental processes in plants (11), but to our knowledge, until now, no study has described the presence of FLPs in viridiplantae. Thus, we addressed the hypothesis that FLPs exist in plants and participate in their physiological responses to hyperosmotic stress. Moreover, since many discoveries with direct relevance to animal biology have been elaborated using plants (17), this topic could also be relevant for metazoan biology by bringing new insight in FLPs structures, functions, and evolution.

## Materials and Methods

### Cell culture conditions

*Arabidopsis thaliana* L. cell suspensions were freshly prepared from calli of the cell line T87 (24), which was generated from the ecotype Columbia plant. They were maintained in Gamborg culture medium complemented with 20 g L^−1^ sucrose, 2 mg L^−1^ 2,4 D, 0.1 mg L^−1^ kinetin at 22 ± 2°C under continuous white light (40 μE m^−2^ s^−1^) with continuous shaking (gyratory shaker at 120 rpm), as previously described (24, 25). Cell suspensions were sub-cultured weekly using a 1:10 dilution. All experiments were performed at 22 ± 2°C using log-phase cells (4 days after sub-culture). Cell density was about 3.10^4^ cells mL^−1^.

### Electrophysiology

Cells were impaled in the culture medium with borosilicate capillary glass (Clark GC 150F) micropipettes (resistance: 50 MΩ when filled with 600 mM KCl). Main ion concentrations in the medium after 4 days were 9 mM K^+^, 11 mM ${\text{NO}}_{3}^{-}$ (26). Individual cells were voltage-clamped using an Axoclamp 2B amplifier (Axon Instruments, Foster City, CA, USA) as previously described (24).

### Hyperosmosis test and cell viability assays

Pretreatments of 15 min with the various plants putative FLPs were done prior to the induction of a hyperosmotic stress by a 400 mM sorbitol exposure (duration: 6 h). Hyperosmosis-induced cell death in the cell suspension culture was determined after staining the dead cells with Evans blue (0.005%, w/v) for 10 min. Cells were counted under a microscope and cells accumulating Evans blue were considered to be dead. At least 500 cells were counted for each independent treatment and the procedure was repeated at least three times for each condition.

### Measurement of intracellular ROS level

For measuring reactive oxygen species (ROS) generation, we used the CellROX^®^ Deep Red Reagent (Molecular probes). The cell-permeant dye is non-fluorescent in a reduced state, and exhibits bright fluorescence upon oxidation by ROS. The cells were pre-incubated for 15 min with 100 μM of peptides and then incubated with 400 mM Sorbitol during 1 h. The cells were incubated with 5 μM CellROX Deep Red for 30 min before recording and then were washed with phosphate-buffered saline buffer. The excitation wavelength was set at 640 nm, and the emission was detected at 665 nm (27). The fluorescence intensity of the cells was measured with a Tecan Infinite 200 Spectrophotometer.

### Seedlings culture

*Arabidopsis thaliana* L. seedlings were grown in an environmentally controlled chamber (8 h photoperiod, under 100 μmol photons m^−2^ s^−1^ at the leaf level, 24 ± 2°C) and plants were weekly watered.

### Preparation of epidermal strips

*Arabidopsis thaliana* leaves from 4 to 6 weeks old plants were harvested 1 h after the beginning of the light period. Epidermal strips were carefully prepared from abaxial epidermis then placed cuticle side-down on microscope slides covered with medical adhesive (Dow Corning 355, Peters surgical) and immediately floated in 10 mM MES pH 6.1, 50 mM KCl, 1 mM CaCl_2_ (opening buffer) under white light (40 μmol photons m^−2^ s^−1^), or in 10 mM MES pH 6.1, 10 mM KCl, 1 mM CaCl_2_ (closing buffer) in dark, for 3 h before future treatments.

### Stomatal aperture measurements

Epidermal strips were analyzed with a Laborlux S (Leica, Germany) microscope (×400). For quantifying, microscope fields were digitalized with a Kappa CF11DSP (Nikon, Japan) digital camera. The width of the stomatal aperture was measured using the image analysis software Metreo Kappa Image Base (Kappa, Germany). The pore width from at least 65 stomata from 2 leaves was measured per treatment and pooled together for statistical analysis. Data are expressed as micrometer and are means ± SE.

### Chemicals

Synthetic peptides (purity >95%) were purchased from Proteogenix (Oberhausbergen, France) and diluted in water.

### *In silico* analysis

Putative plant FLP precursors were detected using a blastp search against the protein sequence database at the NCBI and TAIR, using FMRF–FMRF, FLRF–FLRF, or ILRF–ILRF as query. Only sequences showing more than three repeats were considered. Further sequences were obtained using these results as query using blastp against the UniProtKB database. All sequences were also blasted using tblastn against *A. thaliana* ESTs database at the NCBI and TAIR. When possible, the corresponding genes were localized using the EnsemblPlant database. Some representative sequences are presented in Table 1.

**Table 1:** **Identification of some putative pro-peptides and their genes in *Arabidopsis thaliana***.

Name	Prot id			EnsemblPlants – TAIR10				
	UniProt	gb	gi	Gene localization	EST	Repeats	Transposon	Tandem repeats
F26F24.19	Q9LR27_ARATH	AAF87013.1	9295707	NF	NF	NF	NF	NF
F4N2.6	Q9LQB1_ARATH	AAF27054.1	6730633	1:25976374–25976607	No	Yes	Type II	Yes
F1L3.6	Q9LNR8_ARATH	AAF79458.1	8778450	1:5965755–5966750	TC290437	Yes	Type II	Yes
At2g42050 hypothetical protein	P93742_ARATH	AAB63539.1	1871179	2:17546143–17546559	No	Yes	Type II	Yes
Unnamed protein product (BAB01828)	Q9LS63_ARATH	BAB01828.1	9293925	3:11226216–11226473	No	Yes	Type II	Yes
F22O6_210	Q9SVC3_ARATH	CAB43442.1	4886286	3:19428300–19428917	No	Yes	Type II	Yes
F19F18.60 = At4g37570	Q9SZF1_ARATH	CAB38296.1	4468982	4:17655176–17655730	TC297785	Yes	Type II	Yes
Unnamed protein product (BAB09258)	Q9FGB7_ARATH	BAB09258.1	9758805	5:14153439–14154089	No	Yes	Type I	Yes

*The gene coordinates are given regarding TAIR10 genome assembly (accessible at EnsemblPlants http://plants.ensembl.org/) as chromosome:extend. The EST, repeats, transposon, and tandem are the automatic annotations given during the automatic annotation procedure. NF, not found*.

### Statistics

Significant differences between treatments were determined by the Mann and Whitney test, and *P* values <0.05 were considered significant.

## Results and Discussion

### FLRFa-induced hyperpolarization and ion current regulations in *Arabidopsis thaliana* cells

**Figure 1:**
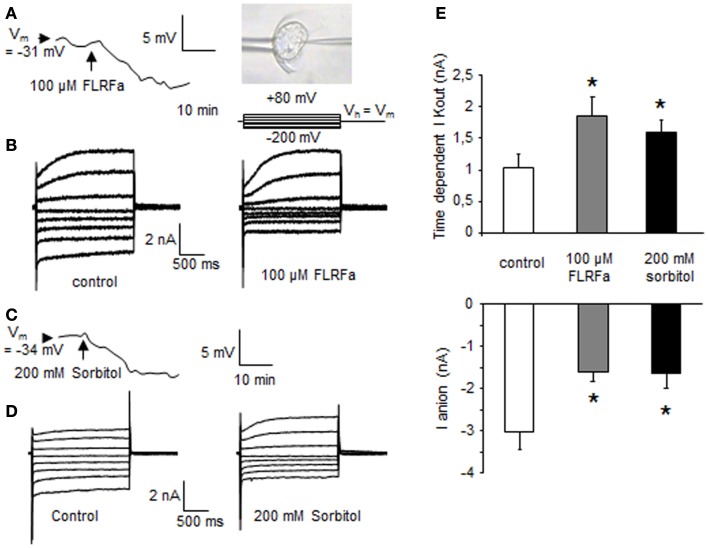
**FLRFa-induced hyperpolarization of a cultured cell of *Arabidopsis thaliana* maintained by a microfunnel and impaled by a microelectrode (A)**. Modulation of *A. thaliana* whole cell currents in response to 100 μM FLRFa **(B)**. Sorbitol induced hyperpolarization **(C)**. Modulation of *A. thaliana* whole cell currents in response to 200 mM sorbitol **(D)**. The protocol was as illustrated, holding potential (V_h_) was V_m_. Mean values of anion currents recorded at −200 mV after 1.8 s and of time dependent K^+^ outward currents at +80 mV after 1.8 s **(E)**. The data correspond to means of four independent experiments and error bars correspond to SD. *Significantly different from controls, *P* < 0.05.

FMRFamide (FMRFa) is a cardioexcitatory peptide that was first isolated from the nervous system of the clam, *Macrocallista nimbosa* (28), and is active as a tetrapeptide only in mollusks and annelids. Other active tetrapeptides have been identified in lophotrochozoans; these include FLRFa, YLRFa, or YMRFa. In view of the well-known effects of FLPs on ion channel regulation in metazoan cells, we first checked for the putative effect of FLRFa, a typical metazoan FLP, on plasma membrane polarization and ion channel regulations in cultured cells of the model plant *A. thaliana* by using single electrode voltage clamp (24). In control conditions (in culture medium), the cell plasma membrane potential (V_m_) of cells was −33 ± 4 mV (*n* = 20) – similar to those we observed in previous studies (25, 26, 29). Addition of 100 μM FLRFa induced a hyperpolarization of the cells of −8 ± 1.5 mV (*n* = 4, Figure 1A). These FLRFa-induced hyperpolarizations were correlated with a decrease in inward currents (Figures 1B,E) that we previously described as anion currents (25, 26, 30), and an increase in time dependent outward rectifying currents (Figures 1B,E), previously described as K^+^ outward currents [KORC, (24, 26, 30)]. It is noteworthy that these ion current regulations are the same as those observed in response to a shift in osmolarity (128–330 mOsm induced by addition of 200 mM sorbitol in the cell culture medium; Figures 1C–E). Inhibition of outward anion currents is a process by which ion leakage is decreased, and thus, results in rapid adaptation to hyperosmotic condition by ion accumulation (31–33). The activation of KORC favoring K^+^ efflux previously reported in other models (34, 35) is opposite to ion accumulation but could be a part of an initial signaling that could result in osmotic regulation (34). In view of these data, we searched for putative plant gene(s) coding for these peptides in gene and protein databases.

### Putative candidate genes for FLPs synthesis in *Arabidopsis thaliana*

**Figure 2:**
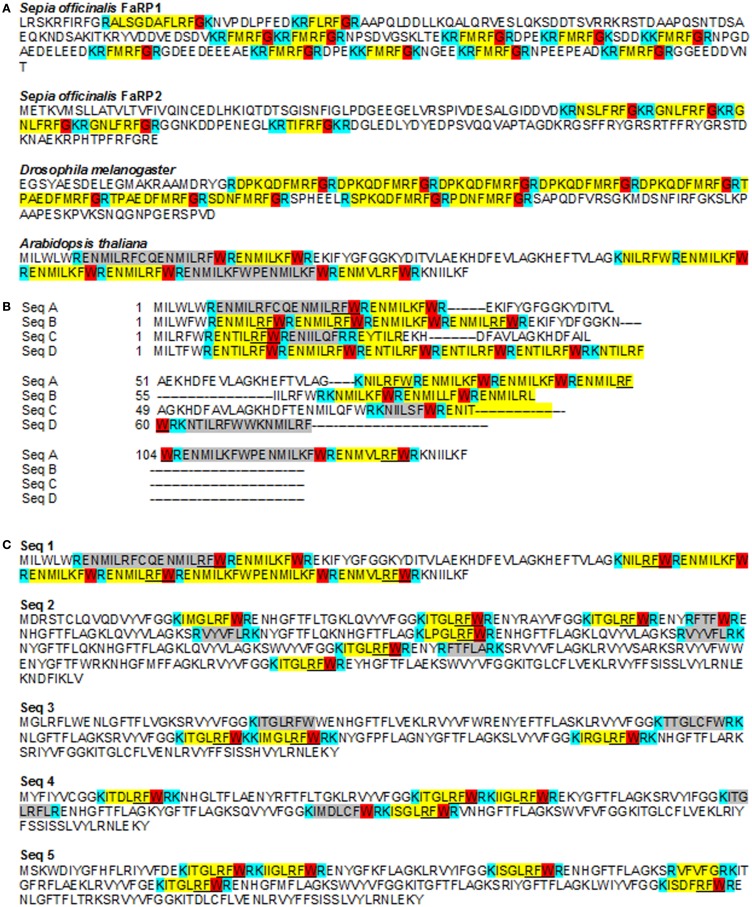
**FMRFamide-like peptides sequences in metazoans and *Arabidopsis thaliana***. **(A)** Partial sequences of FLPs from *Sepia officinalis* (Mollusca), *Drosophila melanogaster* (Arthropoda), and *Arabidopsis thaliana* showing the diversity of FaRP between species, in the same species and in the same gene. For example, two different genes coding for FaRPs were characterized in *Sepia officinalis* with different composition and length. All peptides characterized in metazoans end with a G allowing amidation of the peptide after cleavage (38). The sequence of *A. thaliana* shows similar repetitions ending with RF but with a W instead of a G. Acc number: *S. officinalis* FaRP1: P91889; *S. officinalis* FaRP2: D8WXV2; *D. melanogaster*: AY070639; *A. thaliana*: P93742. **(B)** Different putative pro-peptides in A*. thaliana* genome detected using a poly-ILRF query. Sequences were manually aligned in Jalview. Acc. Numbers: SeqA At2g42050 (P93742_ARATH).1-139; SeqB: BAB01828 (Q9LS63_ARATH)/1-86; SeqC: F26F24.19 (Q9LR27_ARATH_F26F24.19)/1-86; SeqD: F4N2.6_(Q9LQB1_ARATH)/1-77. **(C)** Examples of putative small peptides from *A. thaliana* including a RFW end-sequence (underlined) or terminated by another sequence (highlighted in gray). These sequences are chosen to illustrate the presence of putative pro-peptide genes on each chromosome. Each sequence has been detected in mRNA sequencing (as shown by a tblastn against *A. thaliana* ESTs at the NCBI site) but are not recognized as ESTs in *A. thaliana* EnsemblPlants genomic automatic annotation due to their repeat structure. Instead, all are flagged as transposable elements. Acc. numbers: *A. thaliana*: seq1: P93742_ARATH; seq2: Q9LNR8_ARATH; seq.3: Q9SVC3_ARATH; seq.4: Q9SZF1_ARATH; seq.5: Q9FGB7_ARATH. The putative cleavage sites (mono or dibasic) are indicated in blue, the “transitional peptide” in red, the putative functional peptide in yellow. The sequence highlighted in gray in *Arabidopsis* sequences is a putative peptide.

A FLP can be defined as a peptide that ends in RFa while a FaRP is a peptide homologous to FMRFa in metazoans (36, 37). The number of FLPs identified is increasing with the availability of genome and transcriptome databases and the development of constrained algorithm to search for them (37, 38). The length of these peptides ranges from 4 to 52 amino-acids and 37% of those found do not exceed 10 amino-acids (36). Active peptides are cleaved-out of a pro-peptide; the amino-acids allowing this cleavage are the basic amino-acids K or R, either alone or as a dimer (39) (Figure 2A). In FaRPs, the terminal cleavage site (R/K) is preceded by a G, allowing the amidation of the peptide: the XXRFamide form is biologically active, whereas a non-amidated peptide is considered to be inactive. Regarding FLP, the structure [KR](X)_n_RFG[KR] appears to be the most common organization (Figure 2A). Nevertheless, the amino-acid before the RF ends may vary between peptides and/or organisms. Espinoza et al. (38) have shown that the distribution and the type of amino-acids (the order and the respective position) of each is not random. Although the specificity of each peptide seems to be very high and adapted to each species [see for example (40–42)], the structure and the relationships between structure (composition) and function of the genes, as well as the biochemical characteristic of the couple ligand/receptor, are not clearly established.

By exploring genomic and transcriptomic databases, we found in *A. thaliana*, several putative genes that may allow the production of pro-peptides including repeated FLPs peptide sequences. Some representative sequences are listed in Table 1 and illustrated in Figure 2. As underlined in Table 1, most of these sequences are not annotated as ESTs in the automatic genome annotations, probably because of the “repeat masker” step. Instead, they are interpreted to be transposable elements. But several similar sequences were found in *A. thaliana* ESTs database, suggesting that these genes could in fact be expressed (for example: EG509196 and EG509184). It is noteworthy that the ESTs are issued from *Arabidopsis* stressed with several factors, including salinity, an osmotic stress.

The sequences in Figure 2A were initially identified using a poly-ILRF query and all of them showed the characteristic properties of pro-peptides: tandem repeats and cleavage sites. Each sequence of the alignment in Figure 2B presents 3–12 repeats ending with RF. In these sequences, the IL[RK]F feature is the most abundant. The ILRF sequence is one classical ending identified among the 23 groups of longer metazoan FaRPs (38). Conventional cleavage sites (R or K) are present, suggesting a RENMIL[R,K]FWR peptide sequence. As observed in other genes/species, all the repeats are not followed and/or preceded by a putative cleavage site; these unbreakable repeats are often interpreted as non-functional.

Other repeated peptides with FLPs-like structures have been evidenced. Figure 2C illustrates a group of sequences rich in KI[MIST]GLRFWR and also presenting other putative peptides. Each of these five sequences are found on different chromosomes, indicating that they correspond to different genes.

The peptide ILRF (and YLRF) is found in several combinations of metazoan peptides, suggesting that this peptide could be functional, at least in metazoans (37). This observation, combined with biological effect of tetrapeptide in *Arabidopsis* cells shown above, strongly suggests that functional bioactive FLPs could be synthesized in *Arabidopsis*, as in metazoans. Surprisingly, practically none of the putative peptides observed in *Arabidopsis* ended with a G, but with a W instead. This suggests that in *Arabidopsis*, amidation does not occur when the peptide is generated. This raises the question of the activity of these molecules in *Arabidopsis*, as amidation appears to be necessary to their bioactivity in metazoans.

The putative presence of RF-amide peptides in plants is, at the moment, based on genomic and transcriptomic databases. Future work should aim at characterizing the presence of translated peptides. However, because of the similarity with the metazoan peptides observed in the peptide ILRF found and whatever the differences around the cleavage site, we have explored their activity in *Arabidopsis* cells.

### Putative FLPs from *A. thaliana* could regulate sorbitol-induced PCD and ROS generation

**Figure 3:**
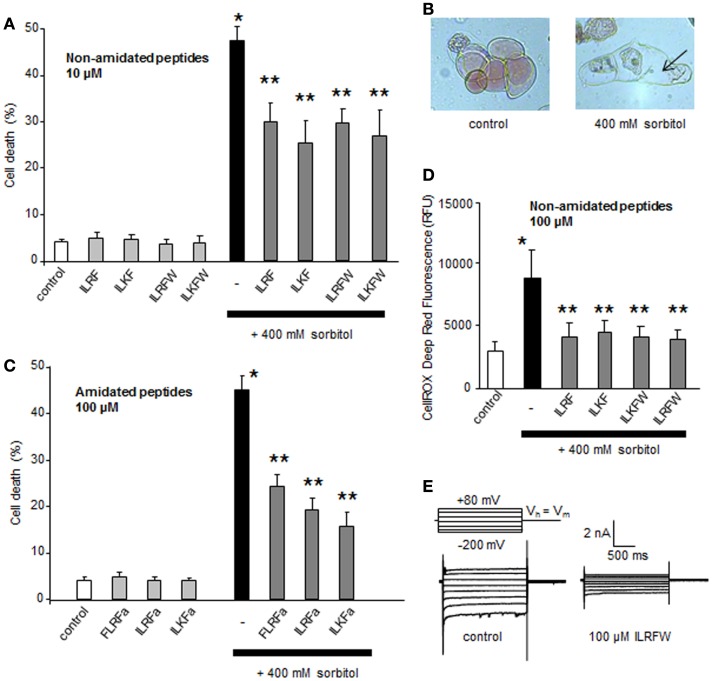
**Effect of different putative plant FLPs on sorbitol-induced cell death in *Arabidopsis thaliana* cells**. **(A)** Effect of non-amidated synthetic FLPs (ILRF, ILKF, ILRFW, and ILKFW, 10 μM each) on sorbitol-induced cell death extent. **(B)** Light micrographs of *A. thaliana* cultured cells stained with Neutral Red 6 h after incubation with 400 mM sorbitol (right) compared to living control cells maintained in their medium (left). Arrows indicate the cell shrinkage. **(C)** Effect of various amidated synthetic FLPs (ILRFa, ILKFa, and FLRFa, 100 μM each) on sorbitol-induced cell death extent. **(D)** Effect of various non-amidated synthetic FLPs (ILRF, ILKF, ILRFW, and ILKFW, 100 μM each) on sorbitol-induced ROS generation. Each data point and error bar reflect the mean and SD, respectively, of at least three independent replicates. *Significantly different from controls, *P* < 0.05 and **significantly different from the sorbitol treated cells, *P* < 0.05. **(E)** Inhibition of *A. thaliana* anion current in response to 100 μM ILRFW.

As in animal cells, plant cell hyperosmotic stress may result in the induction of signaling events that leads to programed cell death (PCD) (2, 43–47), an active cellular process that facilitates the removal of unwanted or damaged cells and is essential for cellular differentiation and tissue homeostasis. We recently showed that hyperosmotic stresses-induced ion channel regulations participate in pathways leading to PCD in plant cultured cells (2). Using synthetic peptides, we tested the effect of putative plant FLPs (ILRF and ILKF, 10 μM each) on sorbitol-induced PCD in *A. thaliana* suspension cells. The shifts in osmolality induced by addition of 400 mM sorbitol (from 128 to 524 mOsm) led to the death of about half of the cell population after 6 h (Figures 3A,B) when FLPs did not induce a significant increase in cell death (Figure 3A). Pretreatments of *A. thaliana* cells with 10 μM ILRF or ILKF 15 min before addition of 400 mM sorbitol decreased the extent of the sorbitol-induced cell death (Figure 3A). Due to the lack of terminal G, which was systematically replaced by a W in plant sequence, the putative plant FLP could not be amidated (cf data from Figure 2). Thus, we further investigated the putative role of the terminal W by testing the same peptides augmented with a terminal W (ILKFW and ILRFW, 10 μM each). These treatments decreased the extent of the sorbitol-induced cell death in the same range (Figure 3A), suggesting no specific role for the terminal W in this response. It is noteworthy that the terminal amidation did not increase the bioactivity, since the decrease in sorbitol-induced cell death by pretreatment with amidated peptides, ILRFa, ILKFa, and FLRFa (100 μM each), were not drastically modified even with a 10 times higher concentration (Figure 3C).

A delayed ${\text{O}}_{2}^{\cdot -}$ generation from NADPH-oxidase activity was also shown to play a central role in the hyperosmotic stress-induced PCD in plant cells (2). We thus evaluated the impact of putative plant FLPs (ILRF, ILKF, ILKFW, and ILRFW, 100 μM each) on sorbitol-induced ROS generation. As observed for sorbitol-induced cell death, pretreatments of *A. thaliana* cells with these FLPs 15 min before addition of 400 mM sorbitol did not cause sorbitol-induced ROS generation (Figure 3D). Moreover, the peptide ILRFW could reduce anion channel activity (Figure 3E) and induce a hyperpolarization of the cells of about −10 mV (not shown) as does FLRFa (Figure 1A). It is noteworthy that non-amidated putative plant FLPs were efficient in decreasing sorbitol-induced ROS generation and anion currents, indicating that the peptide amidation was not necessary for their activities.

From these data it seems that, like in animal cells, FLPs could participate in osmoregulation through regulation of different events as ROS and PCD. The fact that the early FLP-induced ion current regulations in plant cells are the same as the one observed in response to a hyperosmotic stress (Figures 1 and 3E) suggests that FLPs could participate to early induced process to maintain ion homeostasis and/or signalization, allowing to limit hyperosmotic stress-induced-PCD progress (Figure 3A).

### Putative FLPs from *A. thaliana* could regulate stomatal opening

**Figure 4:**
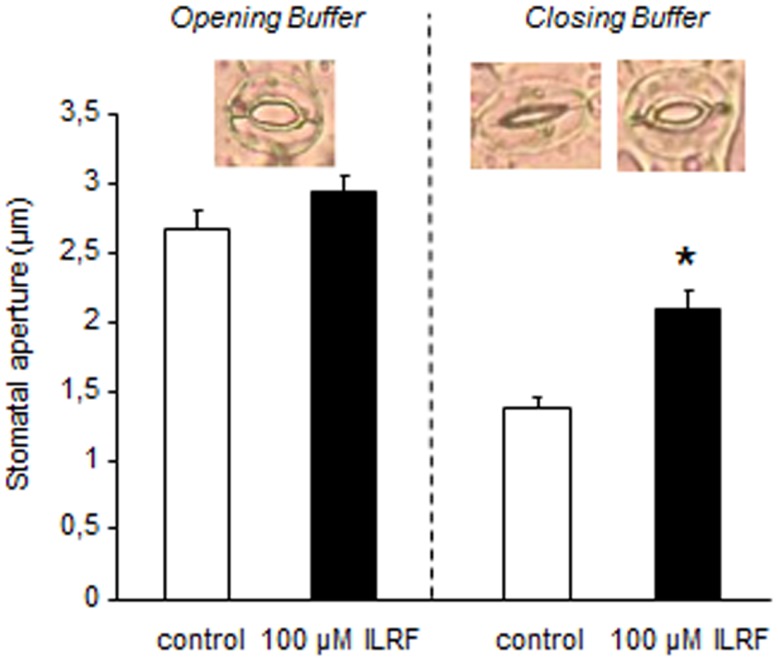
**Changes in *Arabidopsis* stomatal apertures of epidermal strips maintained in opening buffer or closing buffer upon treatment with 100 μM ILRF**. Means ± SE (*n* = 65–75 stomata for each treatment). *Significant difference (*P* < 0.05) in stomatal aperture when compared to the control was found after treatment with ILRF in closing buffer, but not opening buffer.

Since putative plant FLPs could inhibit ROS generation (Figure 3D) and anion channel activity (Figures 1B and 3E) – both of which are known to regulate the stomatal aperture (48, 49) – we checked for the effect of a putative plant FLP (ILRF) on stomatal regulation. Stomata are pores in the plant epidermis that allow gas exchange between the intercellular spaces to the external environment. Two guard cells surround the stomatal pore, and changes in their turgor pressure regulate the size of the pore aperture allowing the CO_2_ assimilation and limit excessive water loss by optimizing the aperture in response to the external environment. Epidermal strips from *A. thaliana* leaves were thus floated 2 h in two different conditions to either optimize the opening (opening buffer) or the closure of the stomata (closing buffer) (29) before addition of 100 μM ILRF. No effect could be observed on open stomata in response to addition of ILRF (Figure 4), while the same treatment induced an increase in the aperture of stomata from the epidermal strips placed in the closing buffer (Figure 4).

This ILRF-induced stomatal opening could appear counterintuitive, since upon drought stress, the stomatal closing is thought to be an important primary defense against tissue dehydration (48, 50). However, if such response is part of a fundamental response to severe drought stress, upon mild drought stress, prolonged closed stomata will stop growth by depriving the plant of CO_2_ for photosynthesis. Adaptive plant growth in sporadic water availability will thus depend on the optimal tradeoff between stomatal closing and rapid re-opening capability. Thus, plants should adapt to mild soil water deficit by mechanisms that are distinct from those of severe dehydration. This was highlighted by a recent report in which acetylated 1,3-diaminopropane counteracted the canonical abscisic acid-induced stomatal closing (48) upon mild drought stress, but not upon severe drought stress (51).

## Conclusion

Numerous cellular features are conserved in eukaryotic cells (18, 19), and many discoveries with direct relevance to animal biology and even human health and disease have been elaborated using plants (17). In this context, conservation of mechanisms of response to hyperosmotic stress, a stress that most of the living organisms have to face, appears logical. In a recent paper, Aalen (10) mentioned that plant peptide research is coming of age and that plant peptide signaling is of crucial importance for all aspects of plant growth and development. Recent works effectively implicate several families of small signaling peptides in various developmental processes in plants (11). However, the families of characterized peptides in plants represent <10% of the estimated number of secreted peptide ligands. Our preliminary results showing that some putative FLPs genes are present in *A. thaliana* genome and that putative plant FLPs could induce physiological responses involved in hyperosmotic stress responses warrant further studies on this topic. Furthermore, we cannot dismiss the possibility that other genes could be responsible for synthesis of others putative FLPs in plants. Drought frequency may increase by more than 20% in some regions of the globe by the end of the twenty-first century, with reductions in crop yields due to decreased water availability. Thus, understanding the putative role of FLPs in plants as regulators that mediate environmental influences on plant development and fitness is particularly relevant for plant biology. Moreover, this topic could also be relevant for metazoan biology since it could bring new insight in FLPs structures, functions, and evolution.

## Conflict of Interest Statement

The authors declare that the research was conducted in the absence of any commercial or financial relationships that could be construed as a potential conflict of interest.

## Abbreviations

FaRPs, FMRFamide related peptides; FLPs, FaRP-like peptides; PM, plasma membrane; V_m_, plasma membrane potential.
